# *In vivo* and *in vitro* therapeutic evaluation of bone marrow-derived mesenchymal stem cells in liver cancer treatment

**DOI:** 10.3389/fcell.2025.1521809

**Published:** 2025-05-08

**Authors:** Abdulrahman Johor, A. S. M. Mahadiuzzaman, Abdulaziz Abdullah Alqusayer, Saleh Abdulaziz Alkarim, F. A. Dain Md Opo

**Affiliations:** ^1^ Department of Biological Science, Faculty of Sciences, King Abdulaziz University, Jeddah, Saudi Arabia; ^2^ Embryonic Stem Cell Research Unit, King Fahd Medical Research Center, King Abdulaziz University, Jeddah, Saudi Arabia; ^3^ Embryonic and Cancer Stem Cell Research Group, King Fahd Medical Research Center, King Abdulaziz University, Jeddah, Saudi Arabia

**Keywords:** *in vivo*, cell culture, 3D sphere, *in vitro*, cell death, tumor

## Abstract

Hepatocellular carcinoma is the seventh most common kind of cancer worldwide and the second largest cause of cancer-related deaths in males, behind lung cancer. Globally, 866,000 people were diagnosed with hepatocellular carcinoma (HCC) in 2022, and nearly 42,240 new cases will be identified in 2025 in the United States. Using stem cells obtained from bone marrow can effectively reduce the number of malignant tumor cells through the induction of an epigenetic impact. We obtained bone marrow-derived mesenchymal stem cells (BM-MSCs) from mice and collected the conditioned medium (CM) from cultured cells with 90% confluency. The effect of the CM was identified using both 2D and 3D sphere cultures of wild-type human liver cancer cell line (HepG2), considering variations in sphere size and percentage. A cell death study was conducted using the cell cytotoxicity (MTT) kit, while the quantity of stem cells was determined by immunohistochemistry and gene expression analysis. The effectiveness of our therapy was demonstrated by an *in vivo* assessment of BM-MSCs through intravenous injection and the currently available anticancer drug cisplatin. *In vitro*, the combination treatment resulted in a synergetic effect, leading to 74% cell death in both adherent and spherical cultures when treated with 25 µM of cisplatin and 90%CM. *In vivo*, the histological study indicated a decrease in tumor size and number following treatment with cisplatin and BM-MSCs. The study lasted 18 weeks and revealed that the body weight of mice improved across all treatment groups, with the combination group exhibiting the most significant improvement. Both *in vitro* and *in vivo* studies showed the synergetic effect of cisplatin and isolated conditioned medium. Our study aimed to identify more efficient therapeutic approaches utilizing stem cells and existing marketed medications to minimize adverse effects with better efficacy.

## 1 Introduction

The fifth most frequent manifestation of cancer in the world today is hepatocellular carcinoma (HCC). In 2022, approximately 866,000 new HCC cases were diagnosed around the world (Liver Cancer Statistics, 2012). After lung cancer, HCC is the second most common cause of cancer mortality among men (Ferlay J et al., 2015). Globally, HCC is the main histological type of liver cancer, accounting for more than 75% of the total number of liver cancers (Petrick JL et al., 2020). It was responsible for 780,000 fatalities in 2018 (Akinyemiju T et al., 2017). With an 18% 5-year survival rate, HCC comes in second to pancreatic cancer (Jemal A et al., 2017). Viral hepatitis (hepatitis B and C), alcoholic liver disease, and non-alcoholic steatohepatitis/non-alcoholic fatty liver disease are all significant risk factors for HCC development (Ioannou GN et al., 2007). Between 2016 and 2030, the incidence of HCC in the United States is anticipated to rise by 122% as obesity and diabetes rates increase (Ioannou et al., 2019). The American Cancer Society predicted that in 2025, nearly 42,240 new cases of primary liver cancer and intrahepatic bile duct cancer will be diagnosed in the United States of America (Key Statistics About Liver Cancer, 20217). To decrease and eliminate hepatic cancerous cells, various types of ongoing research have worked to improve the therapeutic and disease management strategies, including chemotherapy and radiotherapy.

Recently, new studies have demonstrated that mesenchymal stem cells (MSCs) generated from bone marrow (BM) can decrease the epigenetic modification in malignant cells (Lan et al., 2021). MSCs, which are non-hematopoietic multipotent stromal cells with the ability to self-renew, can differentiate into a variety of *in vitro* cell lineages, including adipocytes, chondrocytes, osteocytes, and fibroblasts (Nethi et al., 2023). Bone marrow-derived MSCs (BM-MSCs) are regarded as the major source of isolated stem cells, even though stem cells can also be obtained from a range of sources, including adipose tissue and the umbilical cord (Hong et al., 2014). An essential component of the hepatic malignant environment, mesenchymal stem cells MSCs are thought to play a significant role in the development and spread of tumors (Zhang X et al., 2022). The ability of BM-MSCs to migrate through, attach to, and engraft into the environment of wounded tissues draws attention to their therapeutic potential (Matsuzuka et al., 2010). Several bioactive substances, such as cytokines, growth factors, and extracellular vesicles, which can have immunosuppressive, anti-inflammatory, and apoptotic effects, are released when these engrafted cells are introduced into the tumor microenvironment. To therapeutically activate the anticancer potential, it is possible to introduce BM-MSCs into the hepatic malignant environment.

Cisplatin is a platinum-based drug that is used to treat several solid tumors such as bladder, head and neck, lung, cervical, melanoma, and more than 80% of cancers. In the treatment of HCC, arterial administration of cisplatin combined with systemic therapy produced a synergistic effect. Cisplatin-based chemotherapy is a known treatment against cholangiocarcinoma (CCA) and hepatoblastoma (HB). Induction of necrosis through trans-arterial chemoembolization is a local treatment that contributes to the retention of anticancer drugs by producing an inhibitory effect. Cisplatin usually produces the anticancer effect by inhibiting the G2/M cell cycle phase through DNA damage. This drug was able to arrest the ATM/ATR-Chk1/Chk2 signaling pathways in lenvatinib-resistant HCC (Hamaya et al., 2022). The purpose of our study is to identify the effects of stem cells combined with cisplatin against liver cancer. We performed *in vitro* and *in vivo* experiments to see the effect of the conditioned medium against hepatocellular carcinoma. Using the current study, it is possible to reduce cisplatin resistance by increasing the survival rate with a higher efficacy than the current treatment.

## 2 Materials and methods

### 2.1 Materials

#### 2.1.1 Kits and chemicals

All chemicals and kits used for this research were purchased from Sigma-Aldrich and Thermo Scientific, United States, ensuring high analytical grade. The chemotherapeutic drugs cisplatin (Pt (NH3)2Cl2) and diethylnitrosamine (DEN) were also purchased from Sigma-Aldrich, United States.

#### 2.1.2 Mice and diet

A total of 35 Swiss Albino mice weighing 25–35 gm were purchased and housed in the Animal Laboratory, Department of Pharmacy, King Abdulaziz University, Jeddah, KSA. Mice were allowed to grow in plastic cages (40.5 cm × 25 cm × 15 cm) and kept at 22 ± 1°C and 60% humidity. They were provided with tap water and fed with regular laboratory meals. All mice were monitored daily for signs of toxicity and were weighed three times a week. The animal studies and treatments were approved by the Animal Care and Use Committee of the Faculty of Pharmacy, King Abdulaziz University, Jeddah, KSA.

### 2.2 Methods

#### 2.2.1 Cell cytotoxicity assay

The standard approach for determining the extent to which an inhibitor suppresses cell growth. HepG2 liver cancer cells were plated in 96-well plates and Corning plates for sphere generation and incubated in a CO_2_ incubator for 24 h. Different concentrations of our obtained conditioned medium, commercial drugs, and combinations were applied to the cells for 48 h. The previous culture medium was discarded after being replaced with fresh media bearing MTT chemicals. The plates were incubated for 4 h at 37°C in a CO_2_ incubator until a purple color was developed; the intensity of absorption was quantified at 570 nm with an enzyme-linked immunosorbent assay (ELISA) plate reader. The IC_50_ value was determined using GraphPad Prism.

#### 2.2.2 Collection of conditioned medium

BM-MSCs grew in the adherent cell culture flask and were observed until they reached the confluence of approximately 80% in each flask. Medium without fetal bovine serum (FBS) was provided to each flask kept in the CO_2_ incubator for a specific time. A syringe filter (0.22 µm) was used to collect the CM.

#### 2.2.3 Spheroid cancer cell culture

The HepG2 cells were grown in a cell culture plate, detached by trypsinization, and then centrifuged to get the optimum number of cells. Briefly, 3 × 10^3^ cells were counted and shifted to the 96-well U bottom ultra-low adherent plates (Corning, United States). The plates were centrifuged at 1500 RPM for 5 min and kept in the CO_2_ incubator for 7 days to reach the optimum size. When the spheroid reached 200–260 µm in size at day 8, we introduced our drug (25 µM) and conditioned medium (90%CM) based on the IC_50_ value obtained from the cell cytotoxicity assays. The plates were incubated for 24 h, 48 h, and 72 h on day 9, day 10, and day 11, respectively, in the CO_2_ incubator for morphological analysis. The three wells were utilized for each drug concentration for subsequent morphological and cytotoxicity analyses. A single-sphere cell culture plate was utilized to assess cell cytotoxicity on day 10 following a 48-h treatment and incubation period. The photos were captured utilizing a fluorescence microscope.

#### 2.2.4 Experiment design

The 35 experimental Swiss albino mice models were categorized into five primary groups, and each cohort contained seven animals. The weight and food were monitored each week.

A. Group 1 (Control): This group was prepared as the negative control by providing regular laboratory meals and tap water for seven healthy mice without any treatment.
B. Group 2 (DEN): This group was prepared as the positive control where seven mice were intraperitoneally (IP) injected with carcinogenic chemicals diethylnitrosamine (DEN) to ensure HCC in the mice model following the protocol by Memon et al. (2020). Briefly, each mouse was administrated with DEN in eight separate doses over 4 weeks. All the mice received a dose of DEN twice each week for the development of liver carcinoma. The dosing started with a smaller amount: the initial starting dose was 20 mg of DEN per kilogram of body weight (mg/kg/BW). The second dose was slightly higher, at 30 mg/kg/BW. For the remaining six doses, the amount was further increased to 50 mg/kg/BW for each dose.
C. Group 3 (BM-MSCs): Seven more mice were injected with DEN for liver cancer generation in the BM-MSC group, using the same dosing strategy reported for Group 2. Two weeks after the last DEN injection, these mice were inoculated with an intravenous injection of BM-MSCs mixed in a phosphate-buffered saline (PBS) solution. This injection of BM-MSCs was done three times within the same week, specifically on the second, fifth, and seventh day of that week. To prepare the injection, BM-MSCs were cultured in the PBS solution to ensure 1 × 10^6^ cells in 0.3 mL PBS.
D. Group 4 (Cisplatin): A new cohort of seven mice was administered DEN (diethylnitrosamine) with the identical dosing protocol as outlined for Group 2. After completing their DEN doses, the mice were observed and given a 2-week rest period. Cisplatin was then provided at a dose of 7 mg/kg body weight twice a week to each mouse. Following this rest, the mice were treated with cisplatin delivered *via* IP (IP) on the second, fifth, and seventh days of the same week.
E. Group 5 (Combination): Seven healthy mice were given injections with the carcinogen DEN following the experimental methodology outlined for Group 2. After a 2-week recovery period following the last DEN injection, the mice group was administrated with both cisplatin and BM-MSCs by intravenous injection of 1 × 10^6^ cells three times a week: on the second, fifth, and seventh days of that week. This treatment schedule occurred based on the previously identified protocol (Soleimani M & Nadri S, 2009).

All mice from groups 2 to 5 were euthanized using isoflurane liquid and prepared for further study after 10 weeks of the initial DEN administration. Mice in the control group were also euthanized 10 weeks from the experiment’s start for consistency. After euthanasia, the entire liver of each mouse was carefully removed, with its length and weight recorded. The liver was then divided into sections for further examination and analysis (Tolba R et al., 2015).

#### 2.2.5 Isolation of BM-MSCs

Twenty 8-week-old disease-free mice were prepared for the isolation of BM-MSCs. The mice were rendered unconscious by administering a ketamine/xylazine solution intraperitoneally (1.16 g of ketamine and 2.3 g of xylazine per 1 kg of body weight). The mice model’s tibias, femurs, and humeri were dissected through the experimental period with precise scissors. The bone marrow was removed from the bone cavity by centrifugation process using cell culture media. Separated bone marrows were put through a 70-mm filter mesh to remove unwanted materials. The obtained BM-MSCs were transferred to a cell culture flask and placed inside an incubator set to 37°C and 5% CO_2_ to grow the MSCs. The non-adherent cells were eliminated by changing the old BM-MSC media to the freshly prepared media through 24–72 h of incubation. Cells were sub-cultured by keeping the split ratio of 1:3 when the cultures achieved 70%–90% confluence (Soleimani M & Nadri S, 2009). The presence of BM-MSCs was identified using the specific gene expression by a relative quantitative reverse transcriptase polymerase chain reaction assay. The presence of the stem cells was also identified based on the fluorescence-activated cell sorting (FACS) analysis using the CD34, CD49, and CD90 markers.

#### 2.2.6 Euthanasia process

After setting up an anesthetic chamber with three adsorbents at the bottom, we placed 4.5% of isoflurane with an O_2_ flow rate (1L/min) into the chamber, and once they reached the immobile stage, we introduced compressed CO_2_ gas to the chamber. The chamber was covered with a lid that included a gas inlet and outlet. All the mice were retained for 2–5 min until they were entirely lying down and profoundly euthanatized (de Oliveira, 2020).

#### 2.2.7 Morphological change analysis

At the end of the 18 weeks, all animals from the different groups, including treated and non-treated, were weighed, euthanized by the provided methods, and dissected to obtain the liver. The photographs of the liver were taken using a digital camera placed on the Petri dish. The obtained livers were kept in previously prepared 10% neutral-buffered formalin for further histochemical and immunohistochemical analyses. The remaining fresh liver tissues were collected in cryopreservation tubes and were preserved at −80°C for molecular analyses.

#### 2.2.8 Liver index (%)

The following formula was employed to determine the liver index:

$$\text{Liver Index }\left(\%\right)=\frac{\text{Liver}\;\text{Weight}}{\text{Body}\;\text{Weight}}\times 100\%.$$

#### 2.2.9 Histology and immunohistochemistry of the HCC tissue

In each group of mice, cancer-generated whole liver tissue was collected and opened longitudinally so that it could be examined under a microscope to determine the growth of macroscopic polyps. Four-micrometer thick HCC samples for histopathology were treated in a 4% paraformaldehyde solution and then embedded in paraffin for hematoxylin and eosin (H&E) staining. The number and size of the crypts in the control, DEN-treated, and DEN + MSC-treated livers were counted and compared using H&E staining. Additionally, the SOX2 marker (ab97959, Abcam) was used for immunohistochemistry labeling, which was carried out for microscopy cell proliferation imaging and counting (François et al., 2019). The labeled cells were considered for further analysis by using ImageJ software.

#### 2.2.10 Gene expression analysis

The bone marrow-derived stem cells (BM-MSC) were obtained from mice and cultured for three passages. RNA was isolated for stem cell marker expression analysis and comparison to the culture of the C57BL cell line. Based on the *in vivo* study, the tissue obtained from the sacrificed mice was considered to collect RNA as early as possible. The liver was homogenized for 2 min in a 2-mL Eppendorf tube and the tissue lysate solution was provided by following the protocol (Bioer Technology, China). Following the washing and centrifugation process, the RNA purity was determined using the NanoDropTM spectrophotometer at a wavelength of A260/A280. A RevertAid first-strand cDNA synthesis kit (Thermo Scientific United States, Cat no. EP0451) was used for cDNA synthesis. The expression of several genes (p53, TGF-α, TGF- β, and VEGF) through treatment was measured by quantitative real-time PCR (qRT-PCR) using TB Green TM Premix Ex Taq (TAKARA BIO INC.) following the manufacturer’s instructions. Table 1 shows the human primers used in this study. GAPDH was used as a reference gene. Each sample was run in triplicate. The Livak (2^−ΔΔCT^) method was used to quantify each gene’s expression.

**TABLE 1:** List of primers used for gene expression analysis. The sequence direction was evaluated from the 5^/^ to the 3^/^.

Gene name	Forward	Reverse
P53	5^/^ GTT CCGAGA GCTGAATGA GG 3^/^	5^/^ TTTTATGGCGGGACGTAGAC 3^/^
TGF alpha (TGF-α)	5^/^ AAACACACGAGACGCTGAAG 3^/^	5^/^ ATCCAGTGAGTTCCGAAAGC 3^/^
TGF beta (TGF-β)	5^/^ TACCTGAACCCGTGTTGCTCTC 3^/^	5^/^ GTTGCTGAGGTATCGCCAGGAA 3^/^
VEGF	5^/^ CAG CTATTGCCGTCCAAT TGA 3^/^	5^/^ CCA GGGCTTCATCATTGCA 3^/^
GAPDH	5^/^ GCTCACTAA AGGGCATCCTG 3^/^	5^/^ CCATAGAGGCCATGAGATCC 3^/^

#### 2.2.11 Statistical analysis

The gathered data were analyzed using the Statistical Package for the Social Sciences (SPSS) software, specifically version 22.0.0.0, designed for the Windows operating system. The data are presented in the format of mean ± standard deviation (SD). The data were deemed statistically significant with p-values <0.05, 0.01, and 0.001.

## 3 Results

### 3.1 Morphological study

**FIGURE 1:**
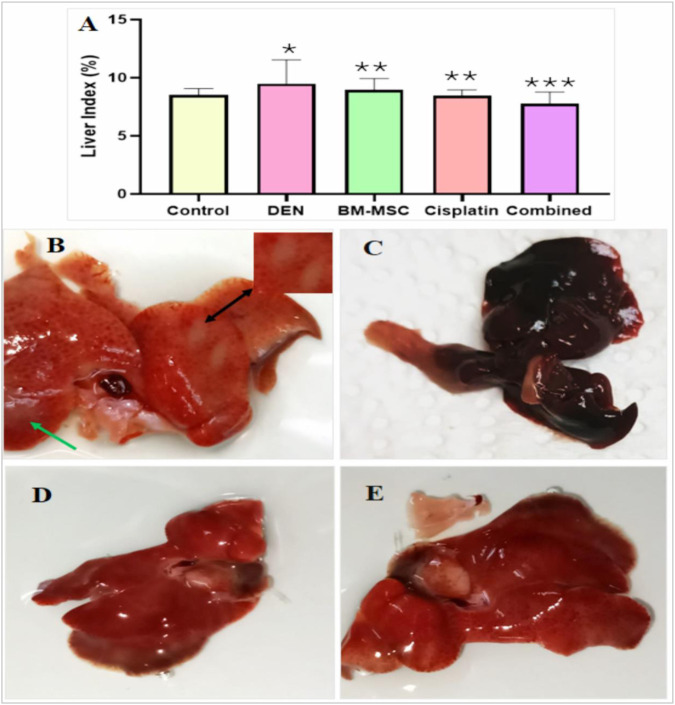
Liver index from all groups **(A)** and the photomacrograph image of four groups of mice including DEN-induced **(B)**, BM-MSCs **(C)**, cisplatin **(D)**, and a combination of BM-MSCs and cisplatin **(E)**. The enlarged inset shows a part of the liver, indicating tumor development **(B)**. Tumor generation (liver discoloration) is indicated by black arrows, and irregular surfaces are indicated by green arrows. The mean difference is indicated statistically at p ≤ 0.05 (*) significant, p ≤ 0.01 (**) highly significant, and p ≤ 0.001 very highly significant (***).

The weights of the livers of all mice were documented immediately after each group of mice was euthanized. The liver index (%) was calculated and statistically analyzed. A large increase in liver index (%) was demonstrated in the DEN-induced mice group compared to those of the treatment groups. The combination treatment with BM-MSCs and cisplatin drug lessened the severity of liver injury compared to the groups treated with only cisplatin or BM-MSCs (Figure 1). The generation of tumors was observed in the DEN-induced group through liver discoloration and an irregular hepatic surface.

### 3.2 BM-MSC isolation and identification

**FIGURE 2:**
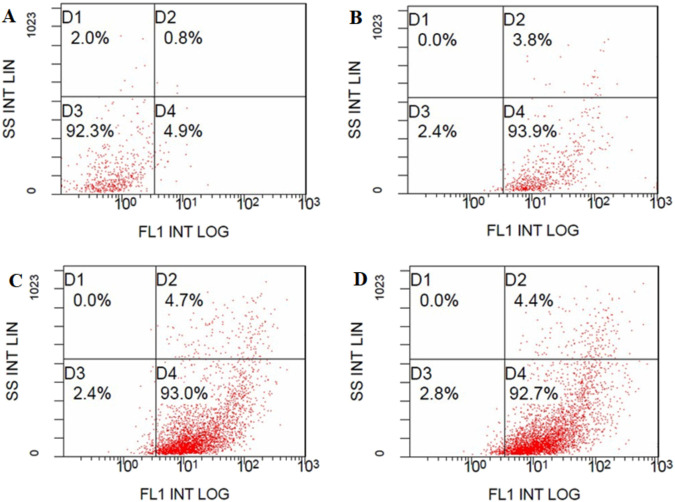
BM-MSC detection using FACS analysis: **(A)** Unstained BM-MSCs (92.3%). BMSC were positive for **(B)** FITC-conjugated CD34 antibody (93.9%), **(C)** FITC-conjugated CD49 antibody (93.0%) and **(D)** FITC-conjugated CD90-IgG antibody (92.7%).

The initial culture of isolated BM-MSCs showed a fibroblast-like shape when observed using the phase contrast microscope. Through the completion of passage three culture, FACS analysis was used to identify the BM-MSC cells on the culture plate. The findings indicated that the MSCs expressed positive levels of CD34, CD49, and CD90 surface markers on more than 90% of cells in contrast to unstained MSCs (Figure 2).

### 3.3 Cell cytotoxicity assay

**FIGURE 3:**
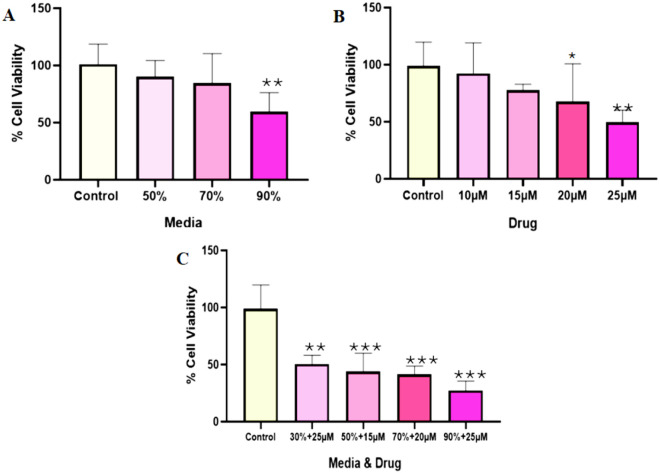
The cytotoxicity analysis on the adherent HepG2 sphere formation. The cells were grown on a plate for 7 days and incubated in a CO_2_ incubator for 48 h based on the treatment. The effects of BM-MSC-derived conditioned medium **(A)**, cisplatin **(B)**, and their combination **(C)** were evaluated across various treatment concentrations. The mean difference was significant at p = 0.05 (*) significantly different from the control group (HepG2), p ≤ 0.01 (**) very significantly different from the DEN group, and p ≤ 0.001 very highly significantly different (***).

The cytotoxicity study of 2D cell cultures showed that cell mortality escalated with rising drug concentrations and while utilizing the conditioned media independently. The drug concentration of 25 µM was associated with the maximum cell death, and the combination of 90%CM and 25 µM cisplatin showed 74% cell death within 48 h of incubation (Figure S1). HepG2 cells were prepared for the 3D sphere cell culture to identify the effect of the selected conditioned medium, drug (cisplatin) alone, or combination of cisplatin and CM. The combination of 90% conditioned medium (CM) with 25 µM drug concentration led to the maximum cell death within 48 h of incubation (Figure 3).

**FIGURE 4:**
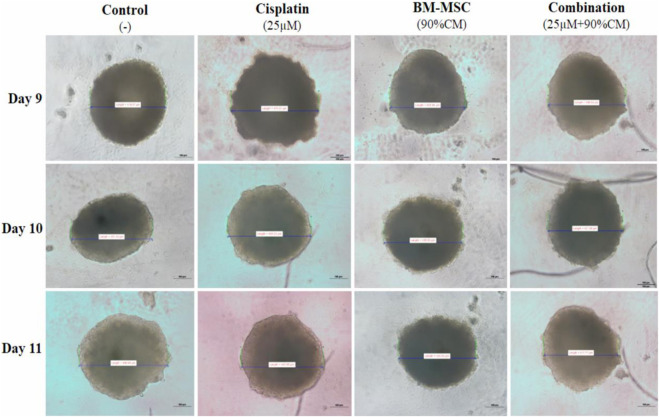
The sizes of the generated HepG2 spheres. For the combination, 90%CM media was used with the 25 µM cisplatin. For the other cases, 25 µM of cisplatin and 90%CM were used separately to treat the sphere. The photos were taken by an inverted microscope.

The morphology analysis of the 3D spheres showed that the size of the spheres decreased through the cisplatin treatment and in the presence of the isolated conditioned medium. The size reduction was observed over the course of the incubation period. The treatments were started with the development of sphere sizes of more than 200–260 µm and proceeded for 72 h of incubation at 37°C in a CO_2_ incubator. The combination treatment approach showed a size reduction of 417.77 µm compared to the HepG2 group alone, whereas the HepG2 sphere size was 496.68 µm through the 72 h of incubation period (Figure 4).

**FIGURE 5:**
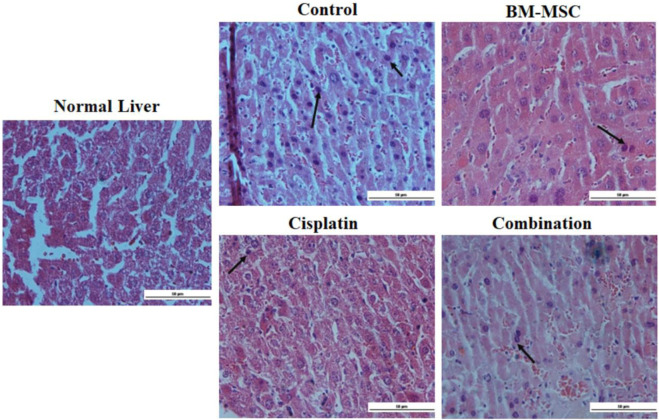
The histopathological analysis of our normal group using a light microscope, DEN-induced liver, and several treated groups (scale bar = 50 µm). Tumor cells with bile production with H&E staining in the DEN-induced group. Black arrows indicate the presence of cancer cells.

The histology analysis showed that the DEN-induced group of mice produced more tumor genesis than the other treatment group, whether treated alone or in combination. The DEN-induced mice group (control) had the highest amount of inflammatory and apoptotic cells. In the DEN-induced group of mice, a significant increase in the size of the liver and nodular hyperplasia was detected in contrast to the normal liver, which showed normal cellular morphology (Figure 5). The BM-MSC, cisplatin, and combination groups exhibited a reduction in the number of cancer cells. Conversely, the combination group demonstrated superior healing compared to the groups that received only BM-MSCs or cisplatin.

### 3.4 Immunohistochemical study

**FIGURE 6:**
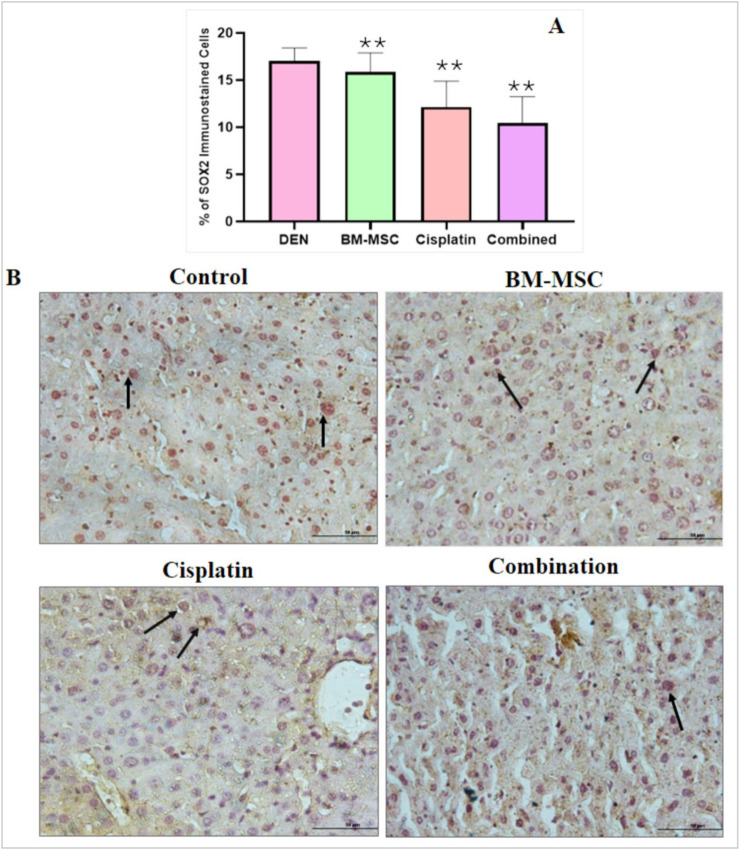
Image of the immunostained sectioned liver with stem cell marker SOX2 for several groups (scale bar = 100 µm). The number of stem cells in the control group **(B)** was relatively high as the control group did not receive any BM-MSCs whereas the treatment groups were injected with a specific number of BM-MSCs. The arrows indicate the presence of cancer stem cells. The percentage of stem cells was calculated using Image J software. The mean difference was significant at level p ≤ 0.05; (*) significantly different from the DEN-induced group (control), p ≤ 0.01, (**) significantly higher different from the control group and p ≤ 0.001 showed a very high significance (***).

The sectioned cells were immunostained with SOX2 antibody, indicating the presence of cancer stem cells in each group. The number of apoptosis cells in the sectioned liver was highest in the DEN-induced group (control). The presence of the BM-MSCs decreased the number of cancer stem cells in the combination group mice, which were able to produce a synergistic effect to cure the induced HCC (Figure 6). The graph shows that the number of cancer stem cells decreased in the case of the combination treatment (Figure 6A).

### 3.5 Gene expression by analysis

**FIGURE 7:**
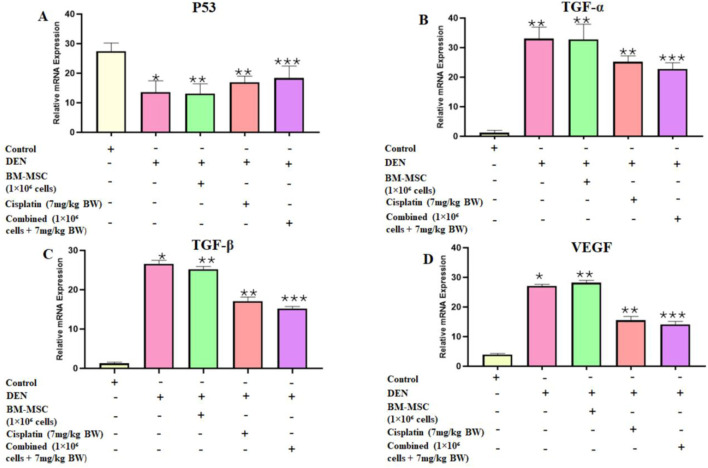
Bar graphs represent the relative expressions of p53 **(A)**, TGF-α **(B)**, TGF-β **(C)**, and VEGF **(D)** at the mRNA level following the analysis of quantitative real-time PCR (qRT-PCR) in the livers of all groups. Data are presented as mean ± SD. The mean difference is indicated as p ≤ 0.05 (*) significant, p ≤ 0.01 (**) highly significant, and p ≤ 0.001 (***) very highly significant compared to the control group. The non-treated healthy mice are considered the control group.

The identification of BM-MSCs was performed through the analysis of CD 90 and CD 44 gene expression. The findings demonstrated that the expression indicators of BM-MSC stem cells were prevalent in the isolated sample (Figure S2). Based on the RNA extraction from mice liver, the individual and combined effects of BM-MSCs and cisplatin on the expressions of p53, TGF-α, TGF-β, and VEGF genes were studied in liver tissues from all groups. The upregulation of p53 in the combined treatment group represented the restoration of diminished expression in the DEN group. On the other hand, in comparison to the BM-MSC- and cisplatin-only groups, the combination treatment of cisplatin and BM-MSCs had a mitigating effect revealed by downregulation of TGF-α, TGF-β, and VEGF expression (Figure 7).

## 4 Discussion

Liver cancer, specifically hepatocellular carcinoma (HCC), constitutes a major global health issue. Conventional treatment modalities encompass surgery, chemotherapy, and radiation therapy; these approaches have limitations regarding efficacy and possible adverse consequences. There has been an increasing interest in the potential of mesenchymal stem cells (MSCs) for treating liver cancer. A recent study reveals a substantial dispute on the pro-tumorigenic *versus* anticancer effects of MSCs. Numerous studies have indicated that MSCs may facilitate tumor growth and metastasis. Conversely, other investigations demonstrated that MSCs inhibit tumor growth contingent upon the specific cancer type and employed animal model (Zong et al., 2018).

Taking these challenges into account, some literature indicated that MSCs could be used to deliver therapeutic agents in the microenvironment of the tumor. Upon homing to the target regions, MSC can release these anticancer drug molecules through the cellular exocytosis mechanism (Mahadiuzzaman et al., 2024). In this study, we used the DEN-induced HCC mice model, which provided multiple treatment doses, including MSCs and chemotherapeutic drugs. In addition, there is a significant interest in the use of MSCs as tumor-specific therapeutic agent-loaded vehicles because of their cellular characteristics, which include tumor tropism, deep migration into the tumor microenvironment, immune evasion, and broad availability and expandability (Szewc et al., 2022). To investigate this, we administered a combined treatment of MSCs and cisplatin drugs to a group of DEN-induced HCC mice.

It has been previously reported that changes in organ weight-to-body weight ratio are a sensitive indicator of acute and subacute drug toxicity (Ong et al., 2016). Based on the organ weight-to-body weight ratio, we calculated the liver index (%), where the DEN group showed a significantly higher toxicity level than the control group. Interestingly, the combined group demonstrated a more significant liver index (%) level than any other group, which represents the effectiveness of the MSC and cisplatin combined treatment approach in HCC.

Inactivation of tumor suppressor gene p53 and activation of proto-oncogene TGF-α and TGF- β result in HCC development. According to research, alterations in p53 have been associated with tumor differentiation, vascular invasion, and tumor stage in HCC. Consistent with previous research, our current results demonstrated that p53 expression was downregulated in the HCC-developed DEN group (Choudhary et al., 2023). Recent research suggests that p53 regulates MSCs mediated tumor suppression in a tumor microenvironment through immune modulation (Huang et al., 2014). Such facts were confirmed in the present study by the simultaneous restoration and upregulation of p53 expression in the MSC group. Furthermore, the combined approach used in our research showed significant upregulation of p53 expression among all other groups.

Inflammatory responses are critically important at various phases of tumor development, such as tumor initiation, promotion, malignant conversion, invasion, and metastasis. Inflammation also influences immune surveillance and therapeutic responses (Grivennikov et al., 2010). The main components of inflammation are chemokines and cytokines. Tumor growth is optimized by the cytokines secreted by cancer cells, while the behavior of malignant cells may be influenced by the cytokines secreted by stromal cells. MSCs can inhibit the growth of liver cancer by secreting cytokines or modulating the cell cycle (Seyhoun et al., 2019). In this study, we investigated the effect of MSCs on three major proinflammatory cytokines, TGF-α, TGF- β, and VEGF, using chemically induced HCC mice models. According to certain studies, TNF-α and TGF-β were reported to induce the expression of VEGF in HCC, which can promote angiogenesis, tumor growth, and metastases (Lucia Pannain et al., 2012; Chen et al., 2019). In our present study, reduced expression of TNF-α, TGF- β, and VEGF was observed in the BM-MSC, cisplatin, and BM-MSCs with cisplatin groups. These findings indicate that MSCs maintained their antitumor and antimetastatic properties when combined with cisplatin.

## 5 Conclusion

Hepatocytes, the primary functional cells of the liver, can differentiate from mesenchymal stem cells (MSCs) into several cell types. This capacity to differentiate has the potential to replace unhealthy or cancerous liver tissue with healthy, functional cells, positioning it as a promising target for liver cancer treatment. Our research showed that the combination of BM-MSCs with cisplatin yielded a synergistic impact, diminishing tumor intensity and cancer progression both *in vitro* and *in vivo*. Additional research is necessary to determine the extent of BM-MSC differentiation into hepatocytes *in vivo*; however, it showed potential therapeutic effects for regenerative therapies.

## Data Availability

The original contributions presented in the study are included in the article/Supplementary Material; further inquiries can be directed to the corresponding author.
