# Supplementary material for: In vivo and in vitro therapeutic evaluation of bone marrow-derived mesenchymal stem cells in liver cancer treatment
Source: Front Cell Dev Biol. 2025 May 8;13:1521809. doi: 10.3389/fcell.2025.1521809 (PMC12095274; doi:10.3389/fcell.2025.1521809)
Supplement: Supplementary file 1 [file DataSheet1.pdf]

Electronic Supplementary File

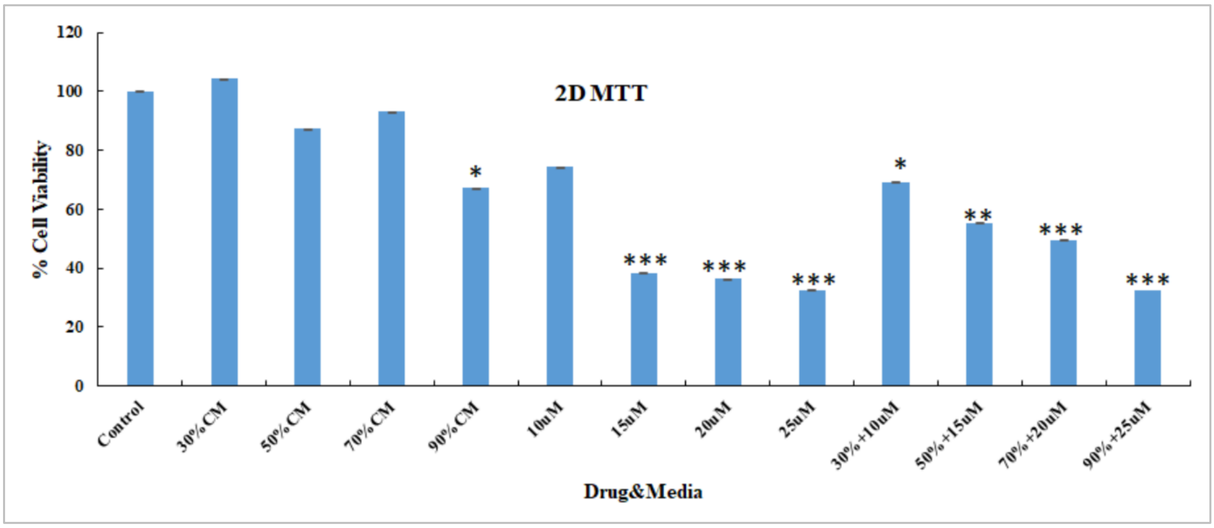

Figure S1. The cell cytotoxicity of the adherent HepG2 cell culture from 2D culture. The cultured cell number decreased with the increasing of time. At the lowest condition medium, the cell growth was predominant in number. Combination with the drugs showed more effective cancer cell death. The mean difference was indicated statistically at level P= 0.05; (*) significant, $p \leq 0.01$, (**) higher significant and $p \leq 0.001$ very high significant (***).

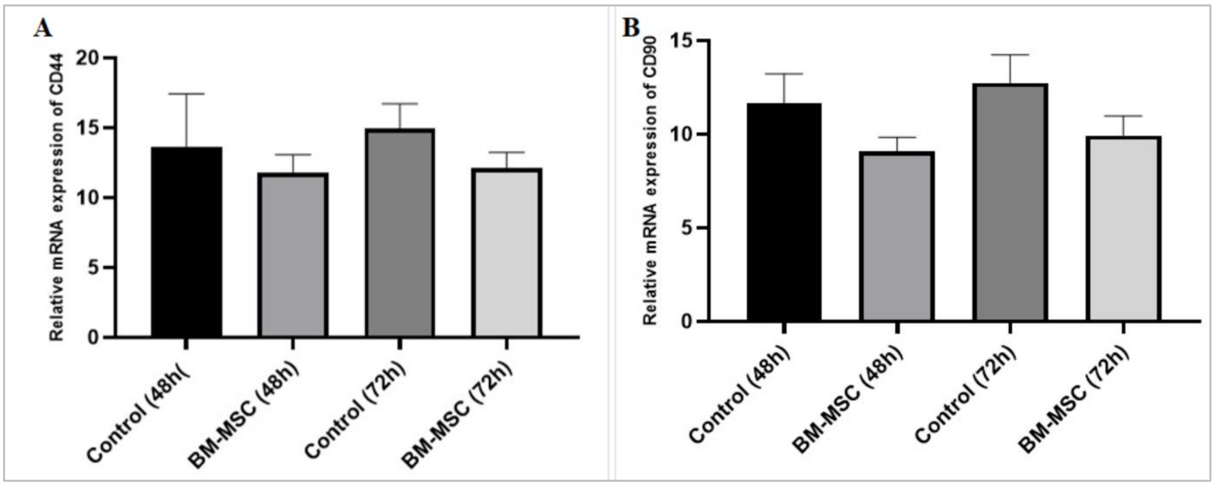

Figure S2. The comparative investigation of the stem cell expression through CD44 (A) and CD90 markers from the extracted RNA acquired from mice BM-MSC and cultivated control stem cells. The control mouse BM-MSC (C57BL) was donated as a gift from King Saud University and grown in a 25 cm^2 cell culture flask that was covered with 0.1% gelatin solution. The RNA was extracted from the mice BM-MSC after passage there and passage six were used to control BM-MSC through the 48 hours and 72 hours incubation.
